# Effects of Dry Needling on Neuromuscular Control of Ankle Stabilizer Muscles and Center of Pressure Displacement in Basketball Players with Chronic Ankle Instability: A Single-Blinded Randomized Controlled Trial

**DOI:** 10.3390/ijerph18042092

**Published:** 2021-02-21

**Authors:** Luis López-González, Deborah Falla, Irene Lázaro-Navas, Cristina Lorenzo-Sánchez-Aguilera, Isabel Rodríguez-Costa, Daniel Pecos-Martín, Tomás Gallego-Izquierdo

**Affiliations:** 1 Department of Physical Therapy, University of Alcalá, 28805 Alcalá de Henares, Spain; luislopezgonzalez4@gmail.com (L.L.-G.); ireneln88@gmail.com (I.L.-N.); cris_lorenzo85@hotmail.com (C.L.-S.-A.); isabel.rodriguezc@uah.es (I.R.-C.); tomas.gallego@uah.es (T.G.-I.); 2 Department of Physical Therapy, University Hospital Ramón y Cajal, 28034 Madrid, Spain; 3 Centre of Precision Rehabilitation for Spinal Pain, University of Birmingham, Birmingham B15 2TT, UK; d.falla@bham.ac.uk

**Keywords:** dry needling, chronic ankle instability, surface electromyography, center of pressure

## Abstract

This study aimed to compare the effects of dry needling (DN) versus placebo DN applied to the peroneus longus (PL) and tibialis anterior (TA) on neuromuscular control and static postural control in basketball players with chronic ankle instability (CAI). A single-blinded randomized controlled trial was conducted. Thirty-two male and female basketball players with CAI were randomly assigned to receive either DN (*n* = 16) or placebo DN (*n* = 16). Pre-activation amplitudes of PL and TA were assessed with surface electromyography (EMG) during a dynamic landing test. Center of pressure (CoP) displacement and sway variability in anterior-posterior (AP) and medio-lateral (ML) directions were measured with a force platform during a single leg balance test (SLBT). Measures were obtained prior to a single DN intervention, immediately after, at 48 h, and 1 month after. The DN group displayed a significant increase in PL and TA pre-activation values, which were maintained 1 month later. Significant reductions in the ML and AP displacements and sway variability of CoP were found for the DN group. These results showed improvements in feedback/feed-forward strategies following DN, including enhanced neuromuscular control and static postural control, with the potential to become a convenient and accessible preventive treatment in CAI subjects.

## 1. Introduction

After an initial ankle sprain, anatomical changes such as laxity, impaired arthrokinematics, or synovial changes can lead to joint insufficiencies that predispose to recurrent ankle sprains [1,2,3,4]. Given the intrinsic characteristics of basketball practice and its specific physical demands, more than 70% of basketball players who suffer an acute ankle sprain will develop recurrent sprains between 6 weeks and 18 months after the initial injury, with approximately 20–40% developing chronic ankle instability (CAI) [1,5,6,7]. CAI is associated with repetitive lateral ankle instability episodes, where both functional and mechanical insufficiencies predispose to multiple sprains [1,4]. CAI is associated with deficits in postural, neuromuscular, and sensorimotor control [5,7,8,9] and is often bilateral [10,11]. 

As a result, a wide variety of altered feedback/feed-forward mechanisms has been shown in subjects with CAI, but it remains uncertain whether these deficits are due to local or spinal/supraspinal conditions [12]. Studies utilising electromyography (EMG) have revealed changes in the activation of the stabilizer muscles of the ankle following injury [5,11,13]. Specifically, these studies have shown lower pre-activation and delayed onset of the peroneal muscles during dynamic tasks in people with CAI [3,10,14,15], which they identified as contributing factors in the etiology of future ankle sprains. Recently, significant differences in preparatory activity displayed by peroneus longus (PL) have been reported in pre-landing scenarios in basketball and volleyball players with CAI, with decreased PL EMG amplitudes just before the foot contact to the ground [16]. In addition, delayed onset of the tibialis anterior (TA) has also been observed in people with CAI [13], as well as an impaired balance in TA-PL co-activation ratio in response to sudden inversion perturbations [17] or pre-landing phases of running and stop-jump maneuvers [18]. 

These changes that occur in people with CAI may be correlated with the presence of myofascial trigger points (MTrPs) [19]. MTrPs are commonly defined as areas of increased irritability located in palpable muscle taut bands and associated with local and referred pain, muscle dysfunction, and autonomic phenomena [19,20]. Although they do not cause spontaneous pain (except when being compressed), latent MTrPS are common both in symptomatic and asymptomatic subjects and can be activated by prolonged exercise or persistent stress of muscle fibers [21,22]. Latent MTrPs may be responsible for leading to accelerated muscle fatigability, poorer control of muscle activity [20,23], and increased sway variability and CoP displacement in medial-lateral (ML) and anterior-posterior (AP) directions during balance tasks, all of the conditions observed in CAI populations [7,10,24,25,26]. Hence, it has been suggested that their “release” may contribute to enhanced sensorimotor function and develop optimal feedback/feed-forward strategies in people with CAI [22]. 

Dry needling (DN) is a technique that aims to diminish persistent peripheral nociceptive inputs by targeting MTrPs [20,23]. Enhanced EMG muscle activation has been observed after DN of latent MTrPs within shoulder muscles [27]. In people with CAI, a combination of PL DN and proprioceptive training led to pain relief and improved perceived functionality [28] as well as to short-term improvements in postural control outcomes [29]. However, to the best of the authors’ knowledge, DN general effects on TA remain to be evaluated. Similarly, the effect of isolated DN on the amplitude of preparatory EMG activity of ankle stabilizing muscles of people with CAI has not been assessed yet. 

Thus, the primary objective of this study was to evaluate the short and medium-term of DN applied on latent MTrPs in PL and TA on EMG pre-activation amplitudes of these muscles immediately before the initial foot contact during a dynamic landing maneuver performed by basketball players with CAI. The second objective was to analyze CoP displacement and sway variability during a single leg balance test (SLBT) as a representative measure of static postural control. We hypothesized that DN would be effective both in the decrease of CoP displacement measures during the SLBT and in the increase of PL and TA pre-activation during the landing maneuver in basketball players with CAI.

## 2. Materials and Methods

A randomized, third-party, single-blinded (subjects and statistician), parallel, controlled trial was conducted following approval from the Committee for Research and Animal Experimentation Ethics of the University of Alcalá, Spain (CEIM/HU/2015/18). The study was prospectively registered in the Australian New Zealand Clinical Trials Registry (ACTRN12616000386437). The study was designed following Consolidated Standards of Reporting Trials (CONSORT) criteria. Ethical principles for clinical research in humans displayed in the Helsinki Declaration were considered. All subjects signed the informed consent and their rights were protected. 

### 2.1. Participant Selection and Randomization

The study population included basketball players aged 18 and over with CAI (Madrid, Spain) meeting selection criteria for the diagnosis of patients with CAI [30] (see Table 1) and presenting with latent MTrPs in both the PL and TA muscles. 

The Spanish Version of the Cumberland Ankle Instability Tool (CAIT) [31] and the Ankle Instability Instrument (AII) [30] were also completed to confirm the presence of CAI. A CAIT score ≤27 and ≥5 affirmative answers to AII confirmed the presence of CAI. When CAI was present in both ankles, the most affected side was assessed and if both ankles were affected equally, the dominant side was chosen. Diagnostic criteria for latent MTrP included: the presence of a hypersensitive spot in a palpable taut band, a palpable or visible local twitch on pincer palpation, and reproduction of referred pain elicited by palpation [20]. Exclusion criteria were: pregnancy; cutaneous lesions or inflammatory edema at the MTrP site; needle phobia; previous adverse reaction to any invasive technique; severe neurological or systemic conditions or lower limb trauma requiring medical treatment during the previous 4 weeks. The subjects were screened for eligibility from March to May 2016.

### 2.2. Sample Size Calculation and Randomization

The sample size was obtained using GPower 3.0.18 software. Considering pre-activation as the primary outcome, an effect size (ES) of 0.25 was considered and the correlation between repeated measurements was assumed at 0.5. By setting four measurements in two treatment groups, sphericity correction determined a total sample size of 24, with a statistical power of 0.80 and an alpha level of 0.05. Considering a potential 20% dropout, 32 patients were recruited (16 per group) [32]. 

### 2.3. Outcomes

At the beginning of the session, the subjects completed a questionnaire to acquire their anthropometric data and exercise habits. 

Measures of EMG (pre-activation) and static postural control (AP and ML displacement and sway variability) (see below) were performed at baseline and then repeated immediately after the intervention, at 48 h, and at one month after the intervention to verify the short and medium-term effects. Exercise practices were controlled for both groups within the time the study was conducted. These measures were obtained by the same physiotherapist, who was not involved in DN treatment. 

#### 2.3.1. Electromyographic Assessment of a Landing Maneuver

**Figure 1:**
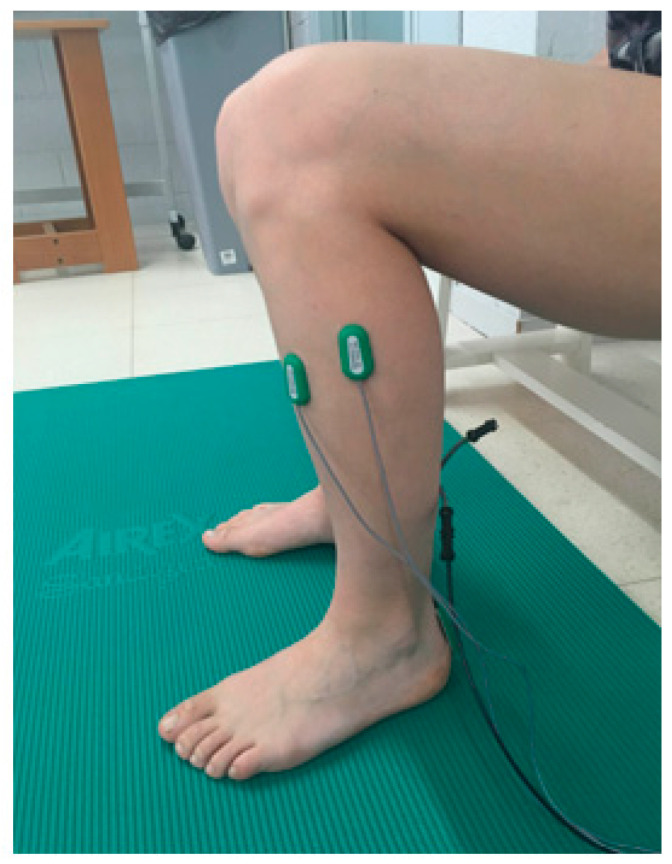
Surface electrode placement.

Prior to electrode placement, the skin was shaved, cleaned, and checked for impedance. Circular 20 × 20 mm pre-amplified bipolar surface electrodes (SX230, Biometrics Ltd. Gwent, UK) were used. With an inter-electrode distance of 20 mm, the electrodes were positioned parallel to the TA and PL muscle fibers following the guidelines for electrode placement (SENIAM recommendations) [33] (Figure 1).

A ground electrode (R506, Biometrics Ltd., Gwent, UK) was placed on the bony prominence of the ulnar styloid. The EMG signals were synchronized with a video file obtained using a video camera Casio EX-FH100 Exilim which permitted obtaining up to 1000 frames per second. A handheld switch with LED (IS3LED, Biometrics Ltd., Gwent, UK) set the start point for the video record and electrical registration and allowed the electrical event to be related to phases of the landing test accurately.

**Figure 2:**
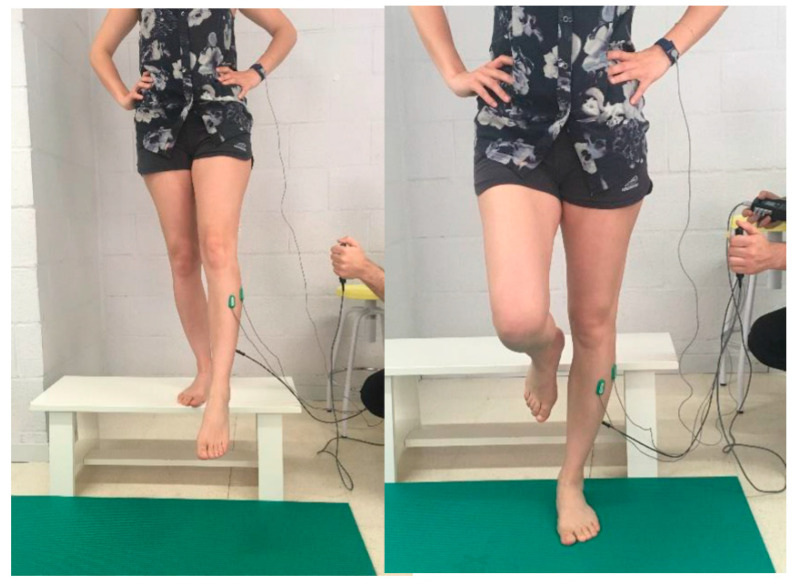
Landing maneuver.

The landing maneuver (Figure 2) to be performed by each player received standardized instructions: players stood barefoot on their unaffected/less affected side on a bench 30 cm high, keeping their eyes on a horizontal line to eliminate visual interference. Their hands were placed over their iliac crests and the limb to be assessed remained lifted, extended, and forward relative to the leg that they were standing. Upon hearing a sound, the participant was asked to land on a horizontal unstable rubber mat 2 cm thick with his forward leg. The first 3 valid attempts in which the participant landed and remained stable on the platform to a maximum total of 5 attempts were considered. Between trials, 30 s of rest were allowed [16].

The preparatory muscle activity (pre-activation) was computed using PC DATALOG Software version 8.51 (Biometrics Ltd., Gwent, UK) and the initial contact was defined as the first video frame in which the foot contacted the ground during the landing task. 

The EMG raw signals were sampled at 1000 Hz and all EMG data were low pass filtered with a common-mode rejection ratio of 110 dB (30 Hz–400 Hz). Then, signals were normalized with respect to peak contraction amplitude averaged from 5 records for each participant, which was obtained before performing the landing maneuver. An analysis window of 200 ms prior to initial contact was set. Then, the analog values of the raw signal were automatically converted into digital values with the Root Mean Square (RMS) analysis, using a time constant of 50 ms. Then, using the RMS values this software calculated the EMG integrated signal, expressed in millivolts/second (mV/s), which permitted to obtain the amount of muscle activity (mV) in the pre-activation phase (200 ms prior to initial contact) [14]. 

#### 2.3.2. Single Leg Balance Test Assessment

The SLBT was carried out immediately after the landing maneuver. A force platform (Kistler Type 5691A1) was used to measure the CoP. First, the weight in Newton (N) of each participant was collected, and subsequently, they stood barefoot in the center of the platform on the affected side, with their knee extended, eyes on a horizontal line, and with hands on the iliac crests. Three measurements, each of 10 s, were obtained. A valid attempt was considered when the participant remained stable without touching the ground with the raised limb during that time. The average of 3 valid attempts was used for the analysis [34].

Bioware 5.1.1.0 (Data Acquisition and InstaCal 6.22) was used to analyze the data. Test result graphs were designed by selecting the graph of displacement in the Ax and Ay axis with respect to measurements of the CoP. In the statistical table, range values in Ax and Ay were taken to establish the AP and ML displacement of the subject’s CoP. The value of the standard deviation was assessed to compute AP and ML sway variability.

### 2.4. Intervention: Dry Needling and Placebo Dry Needling

**Figure 3:**
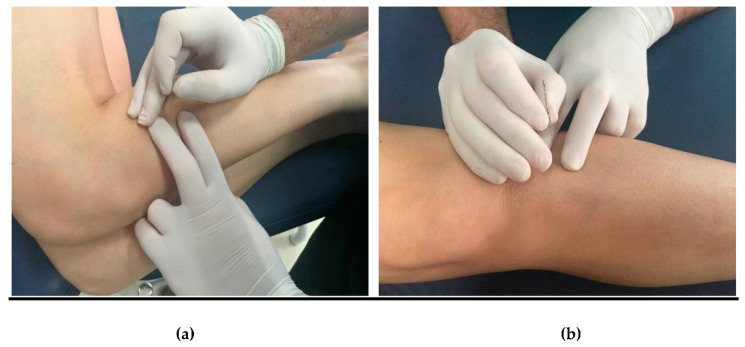
Dry needling of Peroneus Longus (PL) and Tibialis Anterior (TA). (**a**) for PL, the subject was placed in the lateral decubitus position on the non-assessed side with hips and knees flexed to 90 degrees. The physiotherapist remained in front of the patient and inserted the needle perpendicularly from lateral to medial. (**b**) for TA, the subject was placed in supine and the physiotherapist was positioned ipsilateral on the treatment side to guide the needle in an anterior-posterior and slightly medial direction towards the tibia.

Diagnosis of the most painful latent MTrPs of each muscle was determined by a physiotherapist with more than 4 years of experience in the diagnosis/treatment of MTrPs. After shaving and disinfecting the patient’s skin with antiseptic alcohol, the physiotherapist located the most painful latent MTrPs of each muscle and used a needle, sized 0.25 × 0.25 × 50 mm (APS Agu-Punt), for needling (Hong technique) at a frequency of 1 Hz for 30 s (1 puncture per second) [35]. After the first twitch response was obtained, the needle moved vertically 2–3 mm at this frequency [28] (Figure 3). For the placebo group, the same procedure was carried out using placebo needles with the same needle size (Streitberger Placebo—Needle^®^, Asiamed, Pullach, Germany) that did not puncture the skin surface but provoked a needle stick feeling. The handle of these placebo needles was pushed over the needle as soon as it touched the skin, appearing as though the skin was being penetrated, even though it was not [36]. 

### 2.5. Statistical Analysis

Data were analyzed using the Statistical Package for the Social Sciences (SPSS) v.22 software for Windows. All statistical tests were performed considering a *p*-value < 0.05 to determine the effectiveness of the two interventions by the method of intention to treat. The distribution of the data was assessed using the Shapiro-Wilk test. A descriptive analysis of the data for the dependent variables in all measurements was developed: while mean and standard deviation (SD) were calculated for normally distributed dependent variables, medians and interquartile ranges were calculated for non-normally distributed dependent variables.

The homogeneity of the two intervention groups was studied using Student’s T-test for independent samples in the data that were adjusted to the normal and the Mann-Whitney U test for those that did not. For gender, the number of training sessions, training time, and affected side variables the homogeneity were studied using Pearson’s χ^2^ test or Fisher’s exact test, in case that the previous one could not be used.

The design was used to check if the differences in the analyzed variables were due to the treatment, the passing of time, or their interaction, and a general linear model of repeated measures was carried out. The sphericity assumption was checked with the Mauchly test and in the cases that did not meet the sphericity assumption, the Greenhouse-Geisser correction was used. Then the multiple comparisons by Bonferroni correction were applied and the effect size was estimated using the squared Eta parameter (η^2^). Finally, ICCs for all variables considered were calculated applying the two-way mixed model, random effects, and absolute agreement definition

## 3. Results

**Figure 4:**
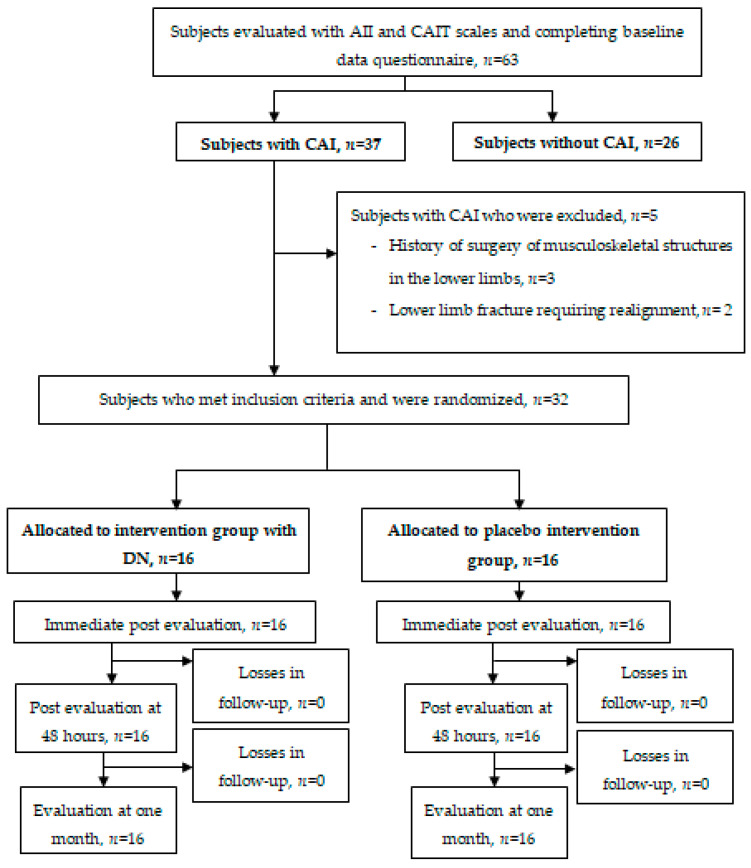
Flowchart of participants. AII = Ankle Instability Instrument, CAIT = Cumberland Ankle Instability Tool, CAI = Chronic Ankle Instability, DN = Dry Needling.

A total of 63 subjects with a history of previous ankle sprains were screened and 31 were excluded as they did not present with CAI or did not meet the selection criteria. Finally, 32 met the established selection criteria (mean age 23.0 [5.0] years; 23 men and 9 women; weight 73.9 kg [11.1]; height 1.8 cm [0.1]; BMI 20.7 [2.6]) and were randomized into two groups: DN (*n* = 16) and placebo (*n* = 16). All participants completed four sessions of assessment and reassessment after treatment. The study flowchart is presented in Figure 4. No significant differences in socio-demographic characteristics (Table 2) and baseline data were found between groups (*p* > 0.05) and no participant reported any adverse event during the study period. ICCs for all variables are presented in Table 3.

### 3.1. Electromyographic Assessment of Tibialis Anterior and Peroneus Longus

The comparison between experimental and control groups (Table 4) for TA and PL pre-activation revealed statistically significant differences for the increase of these variables over time (F_(1,31)_ = 12.716, *p* < 0.001; η^2^ = 0.29 and F_(1,31)_ = 35.468, *p* < 0.001; η^2^ = 0.53 respectively). In both cases the ESs were considered large (Table 4). Within-group differences are presented in Table 5.

### 3.2. CoP Displacement and Sway Variability

Between group differences were found for the decrease of CoP displacement in the ML direction over time, with a large ES in favor of the DN group [F_(1,31)_ = 11.724, *p* < 0.002; η^2^ = 0.27] (Table 4).

Differences for the CoP displacement in the AP direction were also noted and a large ES was obtained for the DN group [F_(1,31)_ = 6.877, *p* < 0.013; η^2^ = 0.18] (Table 4).

Differences in ML and AP sway variability were obtained between baseline and the rest of follow-up measurements [F_(1,31)_ = 16.152, *p* < 0.001; η^2^ = 0.34 and F_(1,31)_ = 8.331, *p* < 0.007; η^2^ = 0.21 respectively]. In both cases, large ESs were displayed. Within-group differences for these variables can be found in Table 5.

## 4. Discussion

After a single session of DN of latent MTrPs within the TA and PL muscles in basketball players with CAI, there was a significant increase in muscle pre-activation noted for PL and TA in favor of the DN group during a landing task (*p* < 0.001). Additionally, statistically significant improvements in static postural control measures (seen as both decreased sway variability and CoP displacement) were achieved in those who received DN (*p* < 0.001), unlike in those who did not. These findings, which were maintained up to one month after the intervention, support changes both in feedback and in feed-forward strategies following DN of PL and TA latent MTrPs.

Neuromuscular dysfunctions and postural control deficits observed in people with CAI have been correlated with the presence of MTrPs after suffering multiple ankle injury episodes [28,37]. In this regard, a sensitivity loss to stretching and a lack of calibration between the intrafusal muscle spindle and its extrafusal fibers are suggested to occur [22]. As a result of this neuromechanical process, diminished accuracy of sensorial inputs (including the proprioceptive) in conjunction with dysfunctional muscle contractions could explain the development of inadequate feedback and feed-forward motor control responses seen in subjects with CAI [38,39]. DN equilibration theory described by Mullins et al. [22] proposed that DN may contribute to improving these motor control responses, by normalizing the intramuscular length-tension relationship followed by MTrP removal, which could lead to muscle spindles sending more optimal afferents to central nervous system. The results of pre-activation amplitude for PL and TA obtained in our study seemed to be consistent with this theoretical framework, reaching statistically significant increases for both muscles (*p* < 0.001) only achieved by the experimental group after latent MTrPs removal, with large ESs (η^2^ = 0.53 and η^2^ = 0.29 respectively).

Similarly, Salom-Moreno et al. [28] obtained increased functionality and decreased pain in people with CAI after applying DN on MTrPs within the PL in combination with 8-week proprioception and specific strengthening programs. Additionally, studies such as those by Rossi [19] or Mullins [29] showed positive immediate or short-term effects after applying DN on PL in CAI subjects, related to improved static and dynamic postural control outcomes. These researchers implied significant progress in the management of CAI athletes with DN, even though neither of them examined EMG changes in neuromuscular behavior following the intervention.

These findings might have important clinical implications given that research has found that in the absence of a direct preparatory stimulus, people with CAI displayed decreased muscle pre-activation and activation values of the entire lower extremity during the unidirectional landing phase when performing a side hop task [40]. In agreement with Ferger et al., other studies have shown poor EMG pre-activation values for PL in CAI subjects during a drop jump and landing tasks, respectively, in comparison to non-CAI subjects [14,16]. In contrast, Hopkins et al. [25] reported greater TA and PL EMG pre-activation in subjects with CAI; however, they focused on the analysis of the initial heel contact during the cycle gait. Literature has shown that motor control adaptations are task-dependent [40] and therefore discrepancies found in Hopkins’ study may be attributed to the different underlying feed-forward mechanisms involved, which might not be comparable to the landing or jumping activities.

Dynamic joint stiffness (DJS) is defined as the joint constraint provided by the musculoskeletal system (ligaments, tendons, or muscles) in dynamic conditions [18]. As with PL EMG activity, our research aimed to include the analysis of TA based on its DJS capacity to resist the injury event and absorb the high ground reaction forces during landing tasks, and an equivalent increase for TA pre-activation was achieved (*p* < 0.001). Whereas previous research has pointed out that lower TA/PL co-contraction indexes (CIs) in the frontal and sagittal planes are common in athletes with CAI [17], these results are opposite to other investigations. Lee and Lin [34] and Tretriluxana et al. [16] found greater TA/PL CIs or non-significant changes respectively in CIs during pre-landing between subjects with or without CAI. As it remains unknown which TA/PL CI values could protect the most against future ankle sprains, we theorize that increased TA and PL pre-activation could translate into more DJS capacity after DN, regardless of possible CIs variations. Future experimental research is needed to sustain our hypothesis and to confirm if these changes correlate with a decrease in the risk of ankle sprain relapses in CAI subjects.

Although changes in feed-forward neuromuscular behavior in the CAI population remain controversial given the heterogeneity of selection criteria, the different tasks considered for EMG analysis, or the choices of EMG measures [41], this study could provide more valuable insight into DN on sensorimotor deficits in CAI conditions. To confirm this statement, this research also aimed to evaluate feedback mechanisms when performing a SLBT after DN. Muscle activity, alpha-gamma co-activation, and supraspinal pathways are hypothesized to be restored after DN and, as a result of enhanced muscle spindle inputs, improved postural control outcomes are expected to occur [22,29].

In this sense, DN also was effective in reducing ML and AP CoP displacement and sway variability of basketball players with CAI, who showed a significant improvement of feedback stabilization strategies in favor of the DN group (*p* < 0.05). These two measures have been identified as some of the most representative metrics of postural control, where larger values are related to lesser stability [42]. Apart from the aforementioned Rossi’s and Salom-Moreno’s conclusions [19,28], our results are again in line with Mullins’ [29] who reported immediate static and dynamic postural control improvements after DN of peroneal muscles. While these improvements were observed both in CAI and control subjects, it must be highlighted that changes were significantly greater in those with CAI [29]. From a clinical point of view, this finding is relevant given that people with CAI display poorer postural control outcomes compared to asymptomatic people [8,13,25,26,41]. Furthermore, systematic reviews have found that impaired postural control is most likely associated with an increased risk of suffering future ankle sprains in people with CAI [43]. Thus, finding complementary approaches to improve postural control, such as DN, could additionally help basketball players with CAI prevent future ankle sprain relapses as well as develop more effective motor control responses during their sports activity.

### Study Limitations

Study limitations included the lack of a control group of asymptomatic subjects to observe the behavior of their EMG activity compared to those with CAI. On the other hand, although statistically significant changes were obtained for the EMG variables and measures of postural control in the DN group, only the most affected limb was investigated and therefore, comparisons with neuromuscular control or postural control of the contralateral limb remain unknown. Additionally, the focus on PL and TA muscles only may limit the conclusions that can be drawn on the effect of DN on neuromuscular control of the lower limb as a whole. Further prospective studies with longer follow-ups are required to understand whether this is a clinically relevant change in terms of the prevention of future ankle injuries.

## 5. Conclusions

The current study provides further knowledge of DN as a treatment of choice in subjects with CAI, showing good results (relative to placebo DN) at increasing pre-activation of ankle stabilizer muscles (PL and TA), both in the short and medium- term. Static postural control also improved following DN, resulting in lower CoP displacement and sway variability in AP and ML directions during a SLBT. These initial results show the potential value of DN in terms of improving feedback and feed-forward strategies, which could be considered within a comprehensive rehabilitation program for people with CAI, inclusive of proprioception and strengthening exercises.

## Figures and Tables

**Table 1:** Selection criteria.

Inclusion Criteria
1. Background history of at least 1 significant ankle sprain. The first sprain must have occurred at least 12 months before inclusion in the research study. It must have developed with signs of inflammation. It must have prevented at least for one day the intended physical activity.
2. Background history of previous sprains, recurrent sprains, and/or feeling of instability. There must have been at least 2 episodes of giving way in at least 6 months prior to inclusion in the study. Recurrent sprain involves 2 or more sprains in the same ankle. Feeling of ankle instability or giving way is defined as “the situation wherein during activities of daily living and sports activities the subject perceives that the ankle joint is unstable and is usually associated with fear of suffering an acute sprain”. The presence of ankle instability must be confirmed through a self-administered questionnaire (Cumberland Ankle Instability Tool).
**Exclusion Criteria**
1. History of previous surgery of musculoskeletal structures in any of the lower limbs.
2. Fracture of any of the lower limbs requiring realignment.
3. Acute musculoskeletal injuries of other joints of the lower limb in the previous 3 months and having interrupted at least one day of the intended physical activity.
4. Having received a specific program for prevention and/or rehabilitation of ankle injuries.
5. Vestibular disorders.
6. Pain in other areas at the time of assessment.

Adapted from Gribble et al., 2014 [30].

**Table 2:** Descriptive characteristics of the control and experimental groups.

Descriptive and Baseline Data	Control Group (*n* = 16)	Experimental Group (*n* = 16)	*p*-Value
Gender (male/female)	11/5	10/6	0.909
Age (years) *	22.06 (4.75)	23.76 (5.23)	0.337
Weight (Kg) *	73.76 (5.98)	74.12 (14.51)	0.927
Height (cm) *	1.78 (0.08)	1.77 (0.06)	0.947
BMI (Kg/m^2^) *	20.64 (1.04)	20.70 (3.47)	0.946
Number of training sessions per week 1,2,3	2/12/2	4/11/1	0.713
Training time per session 1,2,3	0/12/4	1/8/7	0.330
Ankle R/L	7/9	9/7	0.387
CAIT *	21.45 (4.97)	20.75 (5.43)	0.674
AII ^†^	6.50 (4.85;8.13)	6.75 (4.67;8.44)	0.637
Pre-activation TA (mV) *	0.00 (0.00)	0.01 (0.01)	0.873
Pre-activation PL (mV) *	0.02 (0.01)	0.01 (0.01)	0.657
Range ML (mm) *	25.97 (5.78)	27.75 (3.91)	0.305
Range AP (mm) *	24.91(4.48)	25.43 (3.17)	0.704
Sway Variability ML *	5.36 (1.28)	6.12 (1.26)	0.095
Sway Variability AP *	4.68 (0.63)	5.19 (1.13)	0.123

SD = Standard Deviation, BMI = Body Mass Index, R = Right, L = Left, CAIT = Cumberland Ankle Instability Tool, AII = Ankle Instability Instrument, TA = Tibialis Anterior, PL = Peroneus Longus, ML = Medial-Lateral, AP = Anterior-Posterior. No. Training sessions = 1 (2), 2 (3) and 3 (4), Exercise time (minutes) = 1 (30–60), 2 (60–90) and 3 (90–120). * Mean ± SD. ^†^ Median and first and third quartiles.

**Table 3:** The intraclass correlation coefficient (ICC) for all variables.

Variables	ICC	CI 95%	*p*-Value
Sway Variability in AP Direction	0.802	0.63–0.89	<0.001
Sway Variability in ML Direction	0.816	0.66–0.90	<0.001
Range AP	0.835	0.69–0.91	<0.001
Range ML	0.886	0.78–0.94	<0.001
PL Pre-activation	0.964	0.92–0.98	<0.001
TA Pre-activation	0.969	0.96–0.98	<0.001

ICC: Intraclass Correlation Coefficient, CI: Confidence Interval, AP: Anterior-Posterior, ML: Medial-Lateral, PL: Peroneus Longus, TA: Tibialis Anterior.

**Table 4:** Between-group differences.

VARIABLES	GROUP *	Pre	Post	48 h	Month
Pre-activation TA (mV)	Control	0.00 (0.00)	0.00 (0.00)	0.00 (0.00)	0.00 (0.00)
	Experimental	0.01 (0.01)	0.05 (0.00)	0.12 (0.14)	0.09 (0.01)
	Time Interaction per group	**F_(1,31)_ = 12.716, *p* < 0.001; η^2^ = 0.29**			
	Between-group difference in change score ^†,‡^	0.016 (0.009; 0.022) ^§^	0.046 (0.041; 0.051) ^§^		0.088 (0.081; 0.094) ^§^
Pre-activation PL (mV)	Control	0.02 (0.01)	0.00 (0.00)	0.00 (0.00)	0.02 (0.00)
	Experimental	0.01 (0.01)	0.06 (0.01)	0.08 (0.01)	0.06 (0.02)
	Time Interaction per group	**F_(1,31)_ = 35.468, *p* < 0.001; η^2^ = 0.53**			
	Between-group difference in change score ^†,‡^	0.021 (0.014; 0.029) ^§^	0.031 (0.023; 0.038) ^§^		0.034 (0.024; 0.043) ^§^
Range ML (mm)	Control	25.97 (5.78)	27.99 (8.03)	26.69 (5.54)	25.62 (5.46)
	Experimental	27.75 (3.91)	19.51 (3.03)	17.24 (3.23)	14.67 (3.31)
	Time Interaction per group	**F_(1,31)_ = 11.724, *p* < 0.002; η^2^ = 0.27**			
	Between-group difference in change score ^†,‡^	3.108 (0.211; 6.005) *~*	4.980 (1.168; 8.630) *~*		6.715 (3.069; 10.360) ^§^
Range AP (mm)	Control	24.91 (4.48)	24.68 (7.60)	25.02 (4.11)	22.54 (5.54)
	Experimental	25.43 (3.17)	19.29 (1.78)	18.01(1.99)	16.10 (2.28)
	Time Interaction per group	**F_(1,31)_ = 6.877, *p* < 0.013; η^2^ = 0.18**			
	Between-group difference in change score ^†,‡^	3.189 (0.207; 6.172) *~*	3.556 (1.581; 3.5732) ^§^		5.850 (3.465; 8.235) ^§^
Sway Variability ML	Control	4.61 (0.72)	4.54 (0.69)	5.41 (1.0)	5.09 (1.11)
	Experimental	5.84 (0.99)	4.44 (0.89)	3.17 (0.67)	2.52 (1.43)
	Time Interaction per group	**F_(1,31)_ = 16.152, *p* < 0.001; η^2^ = 0.34**			
	Between-group difference in change score ^†,‡^	0.559 (0.185; 0.934) ^§^	0.758 (0.365; 1.520) ^§^		1.242 (0.829; 1.654) ^§^
Sway Variability AP	Control	5.36 (1.28)	5.79 (1.32)	6.00 (1.50)	6.53 (1.81)
	Experimental	6.12 (1.26)	4.31 (0.76)	3.25 (0.89)	2.41 (0.93)
	Time Interaction per group	**F_(1,31)_ = 8.331, *p* < 0.007; η^2^ = 0.21**			
	Between-group difference in change score ^†,‡^	0.691 (0.208–1.175) ^§^	1.114 (0.409–1.819) ^§^		1.270 (0.641–1.898) ^§^

TA = Tibialis Anterior, PL = Peroneus Longus, ML = Medial-Lateral, AP = Anterior-Posterior; * Means and Standard Deviations; ^†^ Compared to pre-treatment; ^‡^ Mean Differences (95% Confidence Interval); ~ Statistically significant differences (*p* < 0.05); § Statistically significant differences (*p* < 0.001); η^2^ = Eta squared. Effect Size.

**Table 5:** Within-group differences.

Variables	Group *	Pre/Post	Pre/48 h	Pre/Month
Pre-activation TA (mV)	Within-group differences ^‡^			
	**F _(1,31)_ = 6.074, *p* < 0.017; η^2^ = 0.16**			
	Control	−0.00 (−0.00; 0.00)	−0.00 (−0.07; −0.006)	−0.00 (−0.00; −0.00)
	Experimental	−0.32 (−0.33; −0.02) ^§^	−0.10 (−0.17; −0.03) ~	−0.07 (−0.08; −0.06) ^§^
Pre-activation PL (mV)	Within-group differences ^‡^			
	**F_(1,31)_ = 49.007, *p* < 0.001; η^2^ = 0.62**			
	Control	0.00 (−0.00; 0.02)	0.01 (−0.00; 0.02)	0.00 (−0.00; 0.02)
	Experimental	0.01 (0.00; 0.03) ~	0.01(−0.00; 0.03)	0.00 (−0.00; 0.02)
Range ML (mm)	Within-group differences ^‡^			
	F_(1,31)_ = 12.578, *p* < 0.001; η^2^ = 0.29			
	Control	−2.02 (−6.18; 2.13)	−0.72 (−6.07; 4.61)	−4.46 (−9.09; 0.16)
	Experimental	8.23 (4.20; 12.27) ^§^	10.50 (5.32; 15.69) ^§^	13.08 (8.59; 15.27) ^§^
Range AP (mm)	Within-group differences ^‡^			
	**F_(1,31)_ = 6.805, *p* < 0.001; η^2^ = 0.18**			
	Control	0.23 (−4.04; 4.51)	−0.10 (−3.08; 2.87)	2.36 (−1.05; 5.79)
	Experimental	6.14 (1.98; 10.29) ^§^	7.42 (4.53; 10.31) ^§^	9.33 (6.01; 12.65) ^§^
Sway Variability ML	Within-group differences ^‡^			
	**F_(1,31)_ = 73.835, *p* < 0.001; η^2^ = 0.70**			
	Control	−0.43 (−1.12; 0.26)	−0.64 (−1.65; 0.36)	−1.17 (−2.60; 2.07)
	Experimental	1.81 (1.14; 2.48) ^§^	2.87 (1.89; 2.48) ^§^	3.71 (2.83; 4.58) ^§^
Sway Variability AP	Within-group differences ^‡^			
	**F_(1,31)_ = 43.401, *p* < 0.001; η^2^ = 0.58**			
	Control	0.01 (−0.50; 0.53)	−0.33 (−0.82; 0.16)	−0.47 (−1.08; 0.11)
	Experimental	1.04 (0.54; 1.54) ^§^	2.30 (1.82; 2.79) ^§^	2.95 (2.38; 3.53) ^§^

TA = Tibialis Anterior, PL = Peroneus Longus, ML = Medial-Lateral, AP = Anterior-Posterior. * Means and Standard Deviations; ^‡^ Mean Differences (95% Confidence Interval); ~ Statistically significant differences (*p* < 0.05); ^§^ Statistically significant differences (*p* < 0.001); η^2^ = Eta squared. Effect Size.
